# Sustainable design of organic solar cells utilized machine and deep learning

**DOI:** 10.1038/s41598-026-35067-7

**Published:** 2026-01-27

**Authors:** Ola M. Mohyeldien, Noha H. El-Amary, Ashraf Al Bardawil

**Affiliations:** Arab Academy for Science, Technology and Maritime Transport, AASTMT, Cairo, Egypt

**Keywords:** Convolutional neural networks (CNN), Layer optimization, Organic solar cell (OSC), Power conversion efficiency (*PCE*), SCAPS-1D, Support vector regression (SVR), Energy science and technology, Engineering, Physics

## Abstract

In this work, an Organic Solar Cell (OSC) with a structure of ITO/PEDOT: PSS/PBDB-T: IT-M/PFN-Br/Al is extensively simulated and optimized. The impact of layer thicknesses and materials on device performance is simulated using a one-dimensional solar cell simulator (SCAPS-1D). The simulation model is first validated using experimental data, and it shows a high degree of alignment. Among the various Electron Transport Layers (ETLs) that are investigated, PFN-Br has the highest Power Conversion Efficiency (*PCE*) of 12.04%. The PFN-Br thickness is shown to be most effective at 5 nm. A simulated *PCE* of 19.50% results from the active layer reaching its optimum efficiency at 300 nm. PEDOT: PSS is the most effective Hole Transport Layer (HTL) with reliable performance at thicknesses ranging from 30 to 100 nm. Due to optical interference, the short-circuit current density ($\:{J}_{sc}$) slightly increases. Additionally, based on structural parameters, *PCE* and open-circuit voltage ($\:{V}_{oc}$) are predicted by using Artificial Intelligence (AI) models, including Convolutional Neural Networks (CNN) and Support Vector Regression (SVR). CNN achieves the highest prediction accuracy in modelling *PCE*, demonstrating its ability to model nonuniform photovoltaic behavior. This combined approach using both detailed simulations and AI‑based prediction doesn’t just make organic solar cells more efficient. It also supports the larger goal of making clean energy more accessible and sustainable. By fine‑tuning device designs, cutting down material waste, and simplifying fabrication, this work can help move the world closer to achieving key Sustainable Development Goals (SDG 7, 9, 12, and 13). In this way, advances in solar technology can play a meaningful role in addressing climate and energy challenges.

## Introduction

Over the last decade, interest in Organic Solar Cells (OSCs) has grown steadily, largely because they offer some clear advantages over traditional silicon-based solar technologies. They’re generally more flexible, lighter in weight, and less expensive to manufacture^1^. Improvements in materials, especially the introduction of non-fullerene acceptors and new donor-acceptor polymers have also played a big role in boosting their Power Conversion Efficiency (*PCE*). OSCs tested in the lab have already surpassed 18% efficiency^2^. To ensure that these solar cells can be widely used in real life, much more work needs to be done to improve the layers inside the cells and how they fit together. Selecting the appropriate Electron Transport Layer (ETL) and Hole Transport Layer (HTL) is a crucial step because they significantly affect the solar cell’s performance^3^. These layers play a vital role in moving electrical charges through the solar cell and helping to prevent them from recombining at the boundaries, which would reduce efficiency. For ETLs materials such as PFN-Br, PCBM, and C60 are commonly used. Each of these offers distinct advantages when it comes to how easily charges can move and how well their energy levels match^4,5^. PEDOT: PSS is commonly used as HTL due to its transparency and outstanding conductance^6^. Other materials like PANI: PSS and P3HT are being investigated^7^. The physical thickness of each layer, especially the active layer, has a big effect on important things like how well it absorbs light, how easily carriers move, and how they recombine^8^. Getting the thickness right requires careful balance. While thicker layers can absorb more light, they may also slow down how charges move through the device. Thinner layers usually allow better charge transport, but they might not absorb enough light to perform efficiently^9,10^.

While physical simulations offer valuable insights into design parameters, the growing complexity of OSC structures necessitates more advanced prediction methods. Recently, there’s been a lot of interest in using Machine Learning (ML) methods like Convolutional Neural Networks (CNNs) and Support Vector Regression (SVR) to handle complex, non-linear behaviors and make performance predictions. These methods first became popular in fields like financial forecasting and time-series analysis, but they’re now increasingly used in scientific research too^11–15^. Unlike traditional models, machine learning techniques learn directly from data and can pick up patterns that standard approaches often miss. Because of this, they’re increasingly seen as a practical option when working with large datasets and complicated parameter settings^16^.

This study takes two approaches to improve organic OSCs: numerical simulation and Artificial Intelligence (AI). The first approach uses SCAPS-1D to simulate the OSC structure based on PBDB-T: IT-M as the active layer. The simulation model is validated by comparing it with published experimental results to confirm its accuracy and reliability. During the simulation phase, the device structure is optimized by systematically modifying ETL, the active layer, and HTL. For both the ETL and HTL, varying material candidates are evaluated to identify the highest performance, followed by a thickness optimization study. The active layer thickness is examined to determine its optimal configuration.

**Fig. 1:**
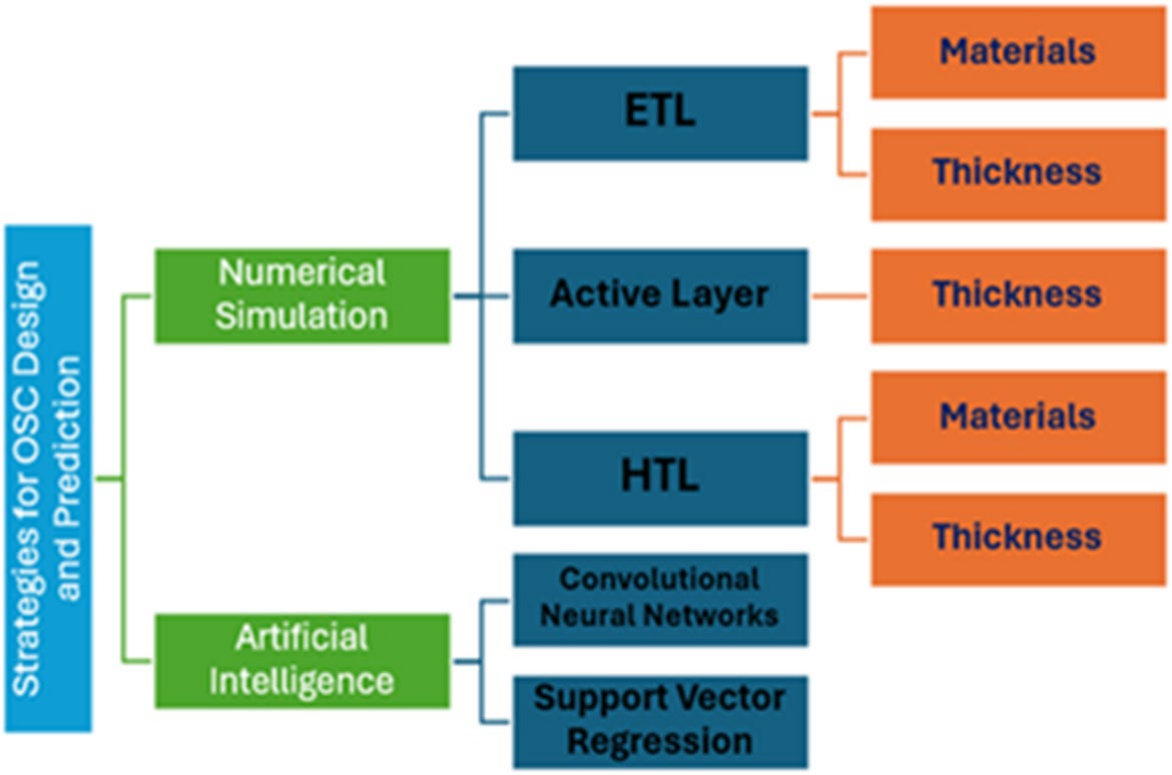
OSC design and prediction strategies.

In the second phase, AI models are created to predict key performance parameters of the designed OSCs. These models are trained on data produced during the simulation step, allowing quick and accurate predictions. This combined approach offers a practical way to speed up the design and improvement of high-efficiency OSCs. Figure 1 illustrates the integration of simulation techniques and machine learning methods aimed at enhancing OSC performance. Moreover, this work goes beyond advancing device performance. It also supports global sustainability priorities. By focusing on high‑efficiency, low‑waste design and aligning with the United Nations Sustainable Development Goals (SDG 7, SDG 9, SDG 12, and SDG 13), this study points out the ability of organic solar technologies to help build a cleaner, more resilient energy future.

## Materials and methods

In this section, a detailed overview of the simulation model, which is used to study the performance of OSCs, is provided. The simulation framework is based on a combination of material properties, device architecture, and computational tools, all of which are carefully optimized to ensure an accurate representation of the device’s behavior under typical operating conditions.

### Device structure

**Fig. 2:**
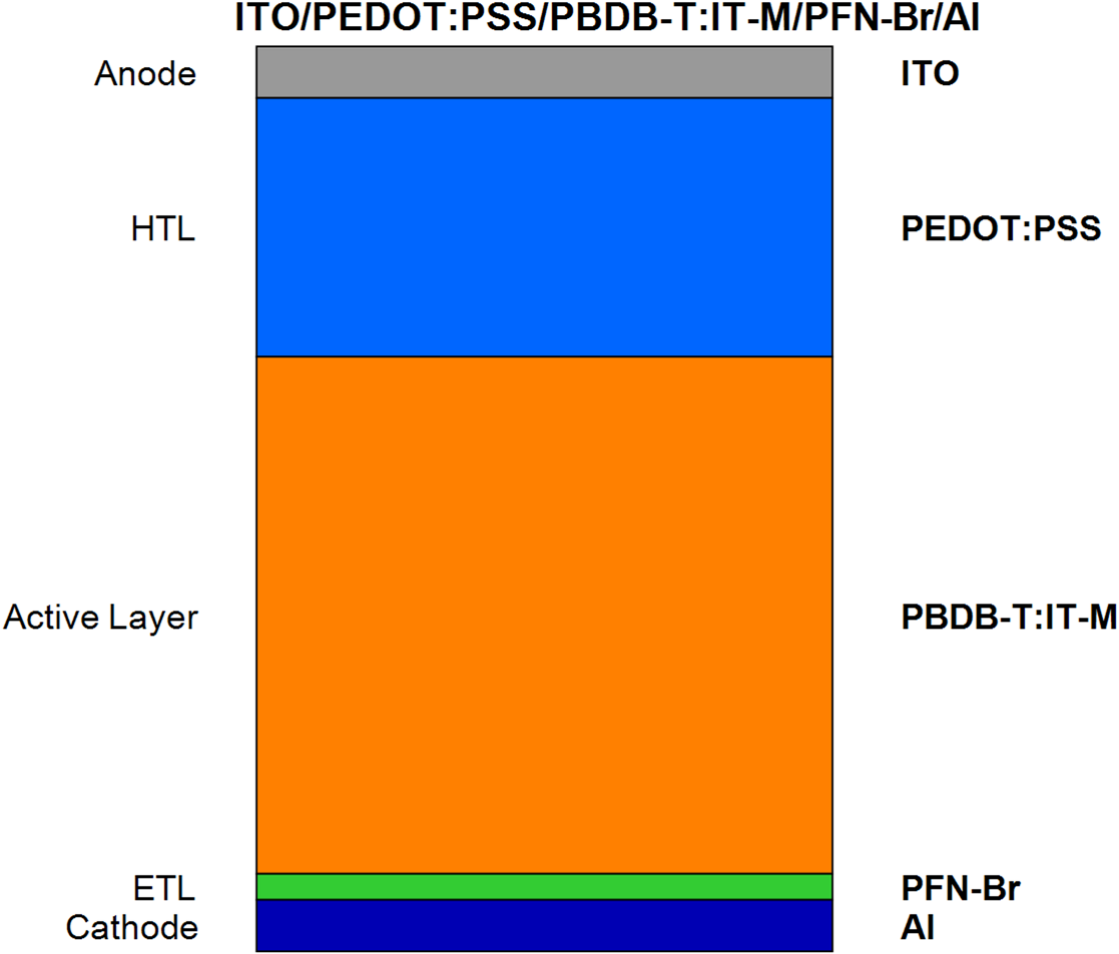
The ITO/PEDOT: PSS/PBDB-T: IT-M/PFN-Br/Al cell structure.

In this study, we use SCAPS-1D, a well-established numerical simulation tool, to simulate the OSC structure for thin-film solar cell modelling. The device architecture under investigation follows the structure ITO/PEDOT: PSS/PBDB-T: IT-M/PFN-Br/Al, as shown in Fig. 2. Indium Tin Oxide (ITO) acts as the transparent conductive electrode, while PEDOT: PSS functions as HTL. PFN-Br serves as ETL with the active layer PBDB-T: IT-M, and aluminum is utilized as the back contact^18^. All the tests are held under standard sunlight conditions (AM1.5G light at 1000 W/m²) and room temperature. Therefore, the results line up with what’s expected in the lab.

### Material parameters

The material parameters used in the simulations are crucial for accurately predicting device behavior. These parameters include thickness, electron affinity, bandgap, effective density of states, and charge mobility of each material in the stack. For the active layer, a widely studied donor-acceptor polymer blend, is chosen due to its promising efficiency in organic photovoltaic devices. In PBDB-T: IT-M device, a low series resistance (< 1 Ω cm^2^) to represent optimized contacts and minimize ohmic losses is employed, together with a high shunt resistance (> 10^3^ Ω cm^2^) to suppress leakage currents. The interface defect density was set to $1 \times {10^{10}}~$ cm^–2^, corresponding to a low-defect interface typically assumed for well-processed thin-film solar cell. Table 1 summarizes the key material parameters used for PEDOT: PSS, PBDB-T: IT-M, and PFN-Br, which form the core of the simulated device.

**Table 1:** Utilized material parameters.

Parameters	PFN-Br^17^	PBDB-T: IT-M^18^	PEDOT: PSS^17^
Thickness (nm)	5	100	40
Bandgap (eV)	2.98	1.2	1.6
Electron affinity χ (eV)	4	4.03	3.4
Relative dielectric permittivity (εr)	5	3.65	3
CB effective DOS ($c{m^{ - 3}}$)	$1*{10^{19}}$	$1*{10^{19}}$	$1*{10^{22}}$
VB effective DOS ($c{m^{ - 3}}$)	$1*{10^{19}}$	$1*{10^{19}}$	$1*{10^{22}}$
Electron mobility µn ($c{m^2}$/V s)	$2*{10^{ - 6}}$	$3.1*{10^{ - 4}}$	$4.5*{10^{ - 5}}$
Hole mobility µp ($c{m^2}$/V s)	$1*{10^{ - 4}}$	$3.2*{10^{ - 4}}$	$9.9*{10^{ - 4}}$
Donor concentration N_D_ ($c{m^{ - 3}}$)	$9*{10^{18}}$	0	0
Acceptor concentration N_A_ ($c{m^{ - 3}}$)	0	0	$2*{10^{18}}$
Thermal velocities V_th_ (cm/s)	$1*{10^7}$	$1*{10^7}$	$1*{10^7}$

### Software and simulation setup

The simulation framework in this study is implemented using SCAPS-1D. It is a one-dimensional numerical simulation tool developed by the University of Gent. SCAPS-1D is extensively used for modelling the electrical behavior of thin-film solar cells, including both organic and inorganic configurations. It numerically solves the fundamental semiconductor equations, which are the electron continuity Eq. (1), hole continuity Eq. (2), and Poisson’s Eq. (3) under steady-state conditions to determine the internal physical processes governing device performance^19,20^:

1 $$\nabla .{J_n}=q\left( {R - G} \right)+q\frac{{\partial n}}{{\partial t}}$$

2 $$- \nabla .{J_p}=q\left( {R - G} \right)+q\frac{{\partial p}}{{\partial t}}$$

3 $$\nabla .\varepsilon \nabla {\mathrm{\boldsymbol{\upvarphi}}}= - {\mathrm{q}}\left( {{\mathrm{p}} - {\mathrm{n}}+{N_D} - {N_A}} \right){\mathrm{~}}$$

where, ${J_n}$ and ${J_p}$ are the electron and hole current densities, n and p represent the electron and hole concentrations, R and G are the recombination and generation rates of charge carriers $\nabla {\mathrm{\boldsymbol{\upvarphi}}}$, is the electrostatic potential gradient, ${N_D}$​ and ${N_A}$​ are the donor and acceptor doping concentrations, respectively.

In the simulation setup, the full device structure, including the interface between each layer and the electrode contacts is considered. Boundary conditions, such as the Schottky or Ohmic contact models for the electrodes, are also implemented to simulate charge collection at the interfaces. The simulation focuses on calculating key photovoltaic parameters. The setup is optimized for steady-state conditions, and the influence of various factors like material properties, layer thickness. The interface quality is explored to predict the optimal performance of the device.

## Results

To validate the accuracy of the simulation model, a comparative analysis is performed between the simulated and experimentally measured photovoltaic parameters of the OSC with the cell structure ITO/PEDOT: PSS/PBDB-T: IT-M/PFN-Br/Al.

**Fig. 3:**
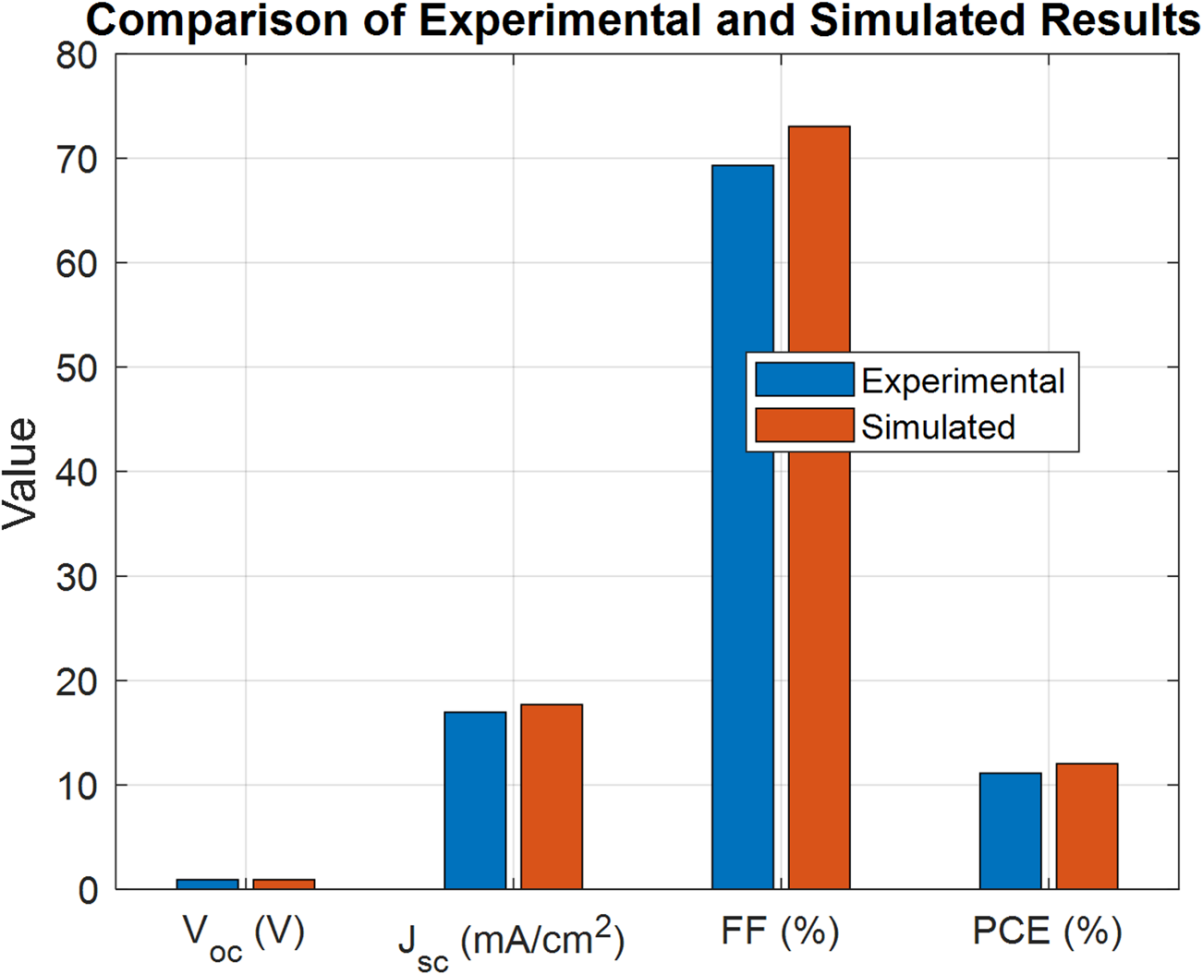
The simulation versus the experimental results.

As shown in Fig. 3, the simulated results demonstrate a strong alignment with the experimental data across key performance metrics as shown in Table 2. The results include ${V_{oc}}$, ${J_{sc}}$, Fill Factor (*FF*), and *PCE*. The close alignment between the simulated and experimental data confirms the reliability of the simulation model. The model predicts a slightly lower open-circuit voltage, decreasing by about 1.5% (0.946 V → 0.9315 V), but this is offset by a modest rise in current density of 4.4% (16.97 → 17.71 mA/cm^2^) and an improvement in the fill factor of 5.4% (69.3% → 73.05%). As a result, the overall efficiency increases by 8.3% (11.13% → 12.05%), confirming that the SCAPS-1D model provides a reliable representation of the device performance. The minor discrepancies observed can likely be attributed to real-world variations, such as material inhomogeneities and device fabrication differences, which are not captured in the idealized simulation setup. These results demonstrate that the simulation model accurately predicts the performance of the organic solar cell, confirming the reliability of the model for further optimization studies.

**Table 2:** The simulation versus the experimental results.

Organic PSC	${{\mathbf{V}}_{{\mathbf{oc}}}}$ (v)	${J_{sc}}~({\mathrm{mA/c}}{{\mathrm{m}}^2})$	FF (%)	PCE (%)
Experimental^10^	0.946 ± 0.003	16.97 ± 0.15	69.3 ± 3.1	11.13 ± 0.53
Simulated	0.9315	17.710396	73.05	12.05

### Results and discussion

This part of the study presents a comprehensive analysis of simulated organic solar cells with the structure ITO/PEDOT: PSS/PBDB-T: IT-M/ETL/Al. The goal is to improve device performance by adjusting key layers within the cell. The analysis focuses on three main aspects: choosing and fine-tuning materials and thicknesses for ETL, examining how different thicknesses of the active layer affect output, and testing HTL materials and their thicknesses. Doing it this way helps highlight which combinations work best during the simulation and gives a clearer picture of what setups are most promising.

#### ETL optimization

To evaluate the impact of ETL on device performance, a series of simulations are conducted using SCAPS-1D, starting with PFN-Br as the reference ETL. The device structure is maintained as ITO / PEDOT: PSS / PBDB-T: IT-M / ETL / Ag, while only the ETL material is varied to isolate its influence. Table 3 summarizes ETL material parameters. The ETLs examined included PFN-Br, PCBM, C60, and PDINN, all of which are organic in nature.

**Fig. 4:**
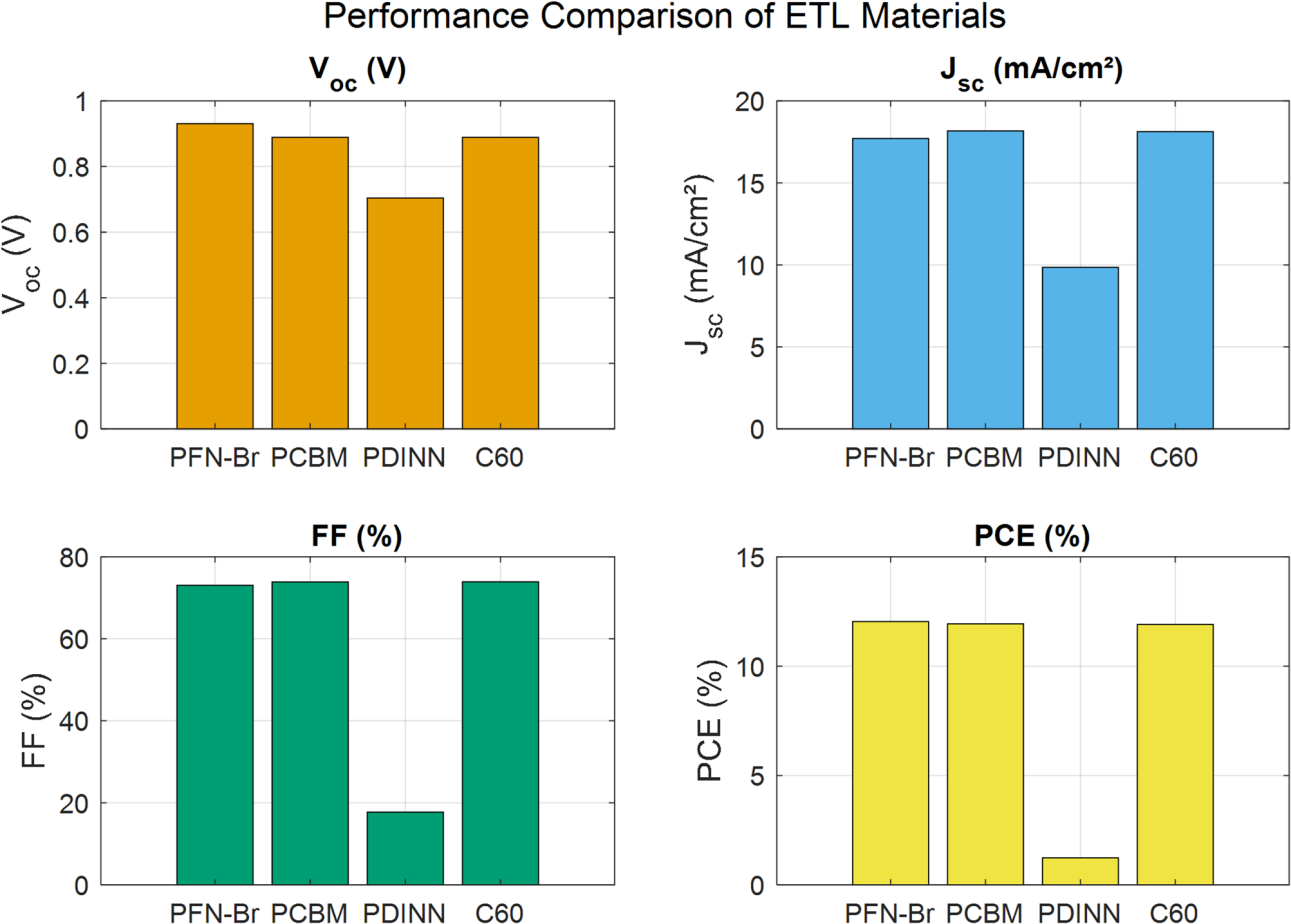
Comparison of the performance parameters for four different ETL materials.

Figure 4 presents a comparison of the key performance parameters for four different ETL materials. Among the tested materials, PFN-Br exhibits the highest *PCE* of 12.04%, with ${V_{oc}}$ of 0.9306 V, ${J_{sc}}$ of 17.71 mA/cm^2^, and *FF* of 73.04%. This superior performance is attributed to PFN-Br’s favorable energy level alignment with the active layer, along with its ability to form low-resistance contact with the silver electrode. PFN-Br is known for its polar functional groups, which modify the work function of the electrode and facilitate efficient electron extraction while suppressing recombination losses^21,22^.

**Table 3:** ETL material parameters.

Parameters	PCBM^22^	PDINN^23^	C60^22^
Thickness (nm)	5	5	5
Bandgap (eV)	2	2.24	1.7
Electron affinity χ (eV)	3.9	3.78	3.9
Relative dielectric permittivity (εr)	3.9	5	4.2
CB effective DOS ($\:{cm}^{-3}$)	$\:2.5*{10}^{21}$	$\:1*{10}^{19}$	$\:8*{10}^{19}$
VB effective DOS ($\:{cm}^{-3}$)	$\:2.5*{10}^{21}$	$\:1*{10}^{19}$	$\:8*{10}^{19}$
Electron mobility µn ($\:{cm}^{2}$/V s)	$\:2*{10}^{-1}$	$\:2*{10}^{-6}$	$\:8*{10}^{-2}$
Hole mobility µp ($\:{cm}^{2}$/V s)	$\:2*{10}^{-1}$	$\:1*{10}^{-3}$	$\:3.5*{10}^{-3}$

In comparison, PCBM and C60 yield slightly lower *PCE* of 11.94% and 11.91%, respectively, with ${V_{oc}}$ values around 0.889 V. Although both materials show higher ${J_{sc}}$ (~ 18.1 mA/cm^2^) and similar *FF* (~ 74%), the modest reduction in ${V_{oc}}$ may result from suboptimal energy level alignment or less effective interfacial dipole formation. Nevertheless, these results are consistent with the known performance of fullerene-based ETLs and affirm their suitability as alternative electron-selective layers^25,26^. The substantial reduction in device performance that is observed with PDINN as the electron transport layer manifested by a low ${V_{oc}}$ (0.7041 V), reduced ${J_{sc}}$ (9.85 mA/cm^2^), and a poor *PCE* of 1.23%, can be attributed to several interrelated factors. Firstly, the energy level alignment between PDINN and the active layer is suboptimal, leading to inefficient electron extraction and reduced built-in potential. Secondly, the electron mobility of PDINN is notably low ($2~ \times ~{10^{ - 6}}$ cm^2^/V s)^24^, which severely limits charge transport and increases the likelihood of carrier recombination. Additionally, PDINN lacks strong dipolar functional groups, resulting in ineffective modification of the silver electrode’s work function and poor interfacial contact. These limitations contribute to increased recombination losses and high series resistance, ultimately suppressing overall device efficiency^27,28^.

#### Thickness optimization of PFN-Br

**Fig. 5:**
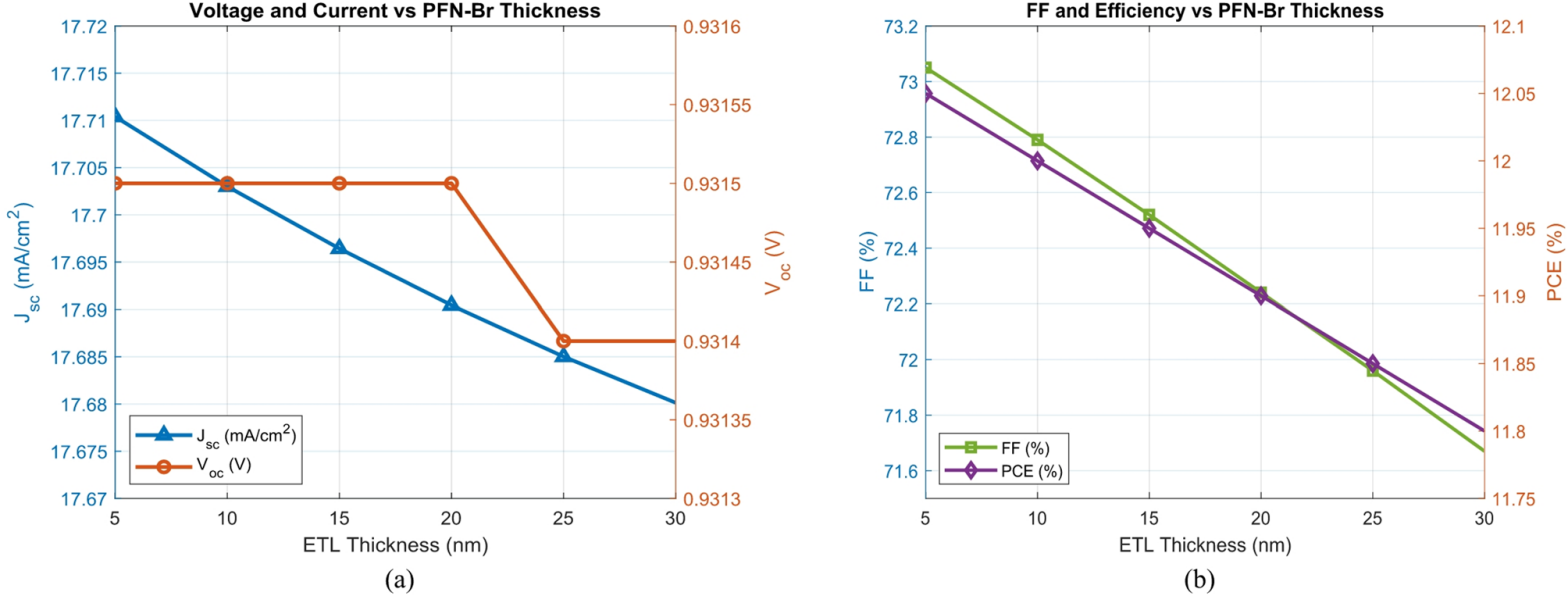
(a) ${V_{oc}}$ and ${J_{sc}}$versus PFN-Br thickness, (b) *FF* and *PCE* versus PFN-Br thickness.

In this section, ETL thickness is varied within the range of 5–30 nm, which is consistent with established practices in high-performance organic solar cells employing thin interfacial layers. As shown in Fig. 5a, the ${J_{sc}}$ exhibits a slight decrease with increasing PFN-Br thickness, from 17.71 mA/cm² at 5 nm to 17.68 mA/cm² at 30 nm. In contrast, ${V_{oc}}$ remains stable at approximately 0.9315 V across all thicknesses, indicating that the energy level alignment and interfacial quality are largely unaffected by the thickness variation. Figure 5b illustrates a more noticeable decline in both the *FF* and *PCE* as the PFN-Br layer thickened. Specifically, *FF* decreases from 73.05% to 5 nm to 71.67% at 30 nm, and *PCE* drops from 12.05% to 11.80%. These trends suggest that increasing ETL thickness leads to higher resistive losses and a reduction in charge extraction efficiency.

When the PFN-Br layer gets thinner, the device seems to work better. Electrons have less distance to cover, so they reach the electrode faster, which cuts down on losses from recombining. Also, with less thickness, the interface might get better, helping charges move more easily. If the layer gets too thin, it might lose its structure or electronic quality. Overall, the results point to 5–10 nm as a good range thin enough to help performance but still stable enough for reliable operation and this aligns with experimental results^29^, showing that such ultrathin layers can indeed be fabricated in practice without compromising device performance.

#### Thickness optimization of PBDB-T: IT-M

In this study, the thickness of the active layer systematically varies from 80 to 400 nm to assess its impact on *PCE*, ${J_{sc}}$, ${V_{oc}}$, and *FF*. This range is chosen due to its prevalence in high-performance OSC architecture and its established potential for optimizing device performance^30^.

**Fig. 6:**
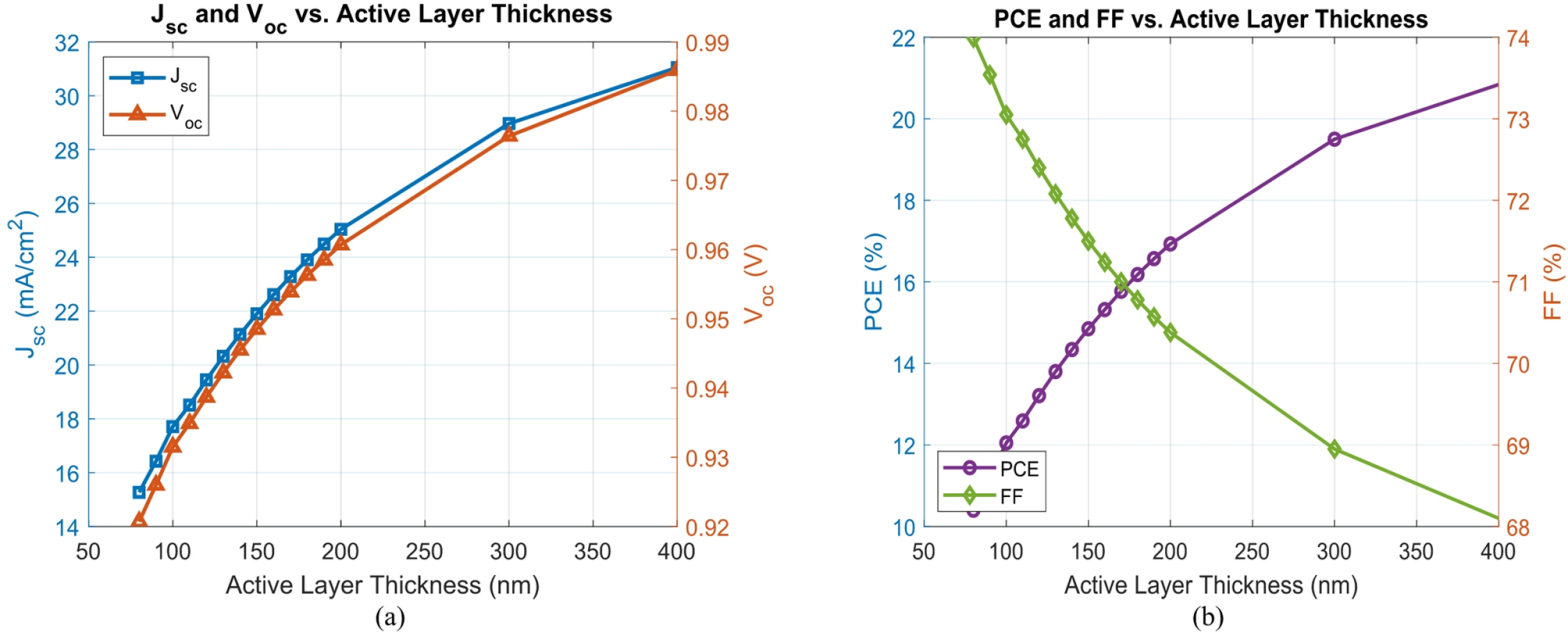
(a) ${V_{oc}}$ and ${J_{sc}}$versus PBDB-T: IT-M thickness, (b) *FF* and *PCE* versus PBDB-T: IT-M thickness.

As shown in Fig. 6(a) and (b), increasing the thickness of the active layer tends to boost light absorption, which helps improve ${J_{sc}}$ and, as a result, raises the *PCE*. For instance, the *PCE* rises from 10.40% at 80 nm to 19.50% at 300 nm. However, although both ${J_{sc}}$ ​ and ${V_{oc}}$ continue to benefit as thickness increases, the *FF* moves in the opposite direction. It drops gradually from 73.99% at 80 nm to 68.10% at 400 nm. This drop suggests that thicker layers introduce greater resistance and encourage more charge recombination, which begins to offset the earlier gains in performance^9^.

Notably, the improvement in efficiency becomes progressively less significant beyond 300 nm. Although a maximum *PCE* of 20.84% is achieved at 400 nm, this improvement is marginal compared to the 300 nm device and comes with significant trade-offs. The *FF* drops further to 68.10%, indicating elevated charge transport losses.

When the active layer gets too thick, the fill factor tends to drop, which points to increased difficulty in moving charge through the material. In practice, it’s not easy to make thick layers uniform during fabrication. They can end up with issues like poor film formation, phase separation, or even cracking. Any of which can reduce how stable the device is over time. Also, charge carriers in organic materials already move slowly, and making the layer thicker just gives them more chances to recombine before they make contact. These limitations outweigh the modest efficiency gains observed at 400 nm. Based on this comprehensive analysis, a thickness of 300 nm is identified as the optimal balance point, offering high optical absorption and superior device performance while maintaining effective charge extraction, manageable resistance losses, and reliable manufacturability. It is clarified that the thickness of the active layer tends to improve ${J_{sc}}$ and, as a result, raises the *PCE*. These optimized dimensions are not only effective in enhancing performance but also readily achievable in practical device fabrication^29^.

#### Influence of hole transport layer (HTL) material and thickness on device performance

To evaluate the role of HTL in OSC performance, both its material composition and thickness are systematically investigated. All tests are conducted within the same device architecture based on the structure ITO/HTL/PBDB-T: IT-M/PFN-Br/Al.

##### HTL material

Three HTL materials are compared: PEDOT: PSS, PANI: PSS, and P3HT, each implemented with a 40 nm thickness. Table 4 summarizes the material parameters used as HTL. demonstrate that PEDOT: PSS outperformed the other candidates, delivering the highest *PCE* of 19.50%, ${J_{sc}}$ of 28.96 mA/cm², and a ${V_{oc}}$ of 0.9764 V. These results are attributed to the favorable energy level alignment and superior hole mobility of PEDOT: PSS, which facilitates efficient charge extraction at the HTL/active layer interface. In contrast, while PANI: PSS exhibited a slightly higher ${J_{sc}}$ (29.46 mA/cm²) and an *FF* of 74.64%, its ${V_{oc}}$ dropped significantly to 0.8577 V, resulting in a lower *PCE* of 18.86%. This reduction in ${V_{oc}}$ may stem from less optimal energy alignment or increased recombination at the HTL interface. The poorest performance is observed for P3HT, which delivered a ${V_{oc}}$ of only 0.8201 V and an *FF* of 56.25%, yielding a *PCE* of 13.29%. These results underline the limited charge transport capabilities and poor interface compatibility of P3HT.

**Table 4:** The material parameters used as HTL.

Parameters	PANI: PSS^31^	P3HT^32^
Thickness (nm)	40	40
Bandgap (eV)	1.2	2.1
Electron affinity χ (eV)	3.7	3.5
Relative dielectric permittivity (εr)	9.5	4.4
CB effective DOS ($c{m^{ - 3}}$)	$1*{10^{19}}$	$2.2*{10^{18}}$
VB effective DOS ($c{m^{ - 3}}$)	$1*{10^{19}}$	$1.9*{10^{19}}$
Electron mobility µn ($c{m^2}$/V s)	1.3	$4.8*{10^{ - 4}}$
Hole mobility µp ($c{m^2}$/V s)	1.3	$4.8*{10^{ - 3}}$

##### HTL thickness optimization

To assess the influence of HTL thickness on device performance, the thickness of the PEDOT: PSS layer varies from 30 to 100 nm, while keeping the rest of the device configuration unchanged.

The *PCE* remains relatively constant across the studied thickness range, increasing slightly from 19.50% at 30 nm to 19.52% at 100 nm. This stability highlights PEDOT: PSS’s tolerance to moderate variations in thickness. The ${V_{oc}}$ also remains virtually unchanged (~ 0.9763–0.9764 V), further supporting the robustness of the energy level alignment at the HTL/active layer interface^33^.

The ${J_{sc}}$, however, exhibited a gradual increase in thickness from 28.92 mA/cm² at 30 nm to 29.22 mA/cm² at 100 nm likely due to improved optical interference and light trapping effects. In contrast, the *FF* showed a minor but consistent decrease from 69.04% to 68.42%, potentially resulting from increased series resistance associated with thicker PEDOT: PSS layers^34^.

**Fig. 7:**
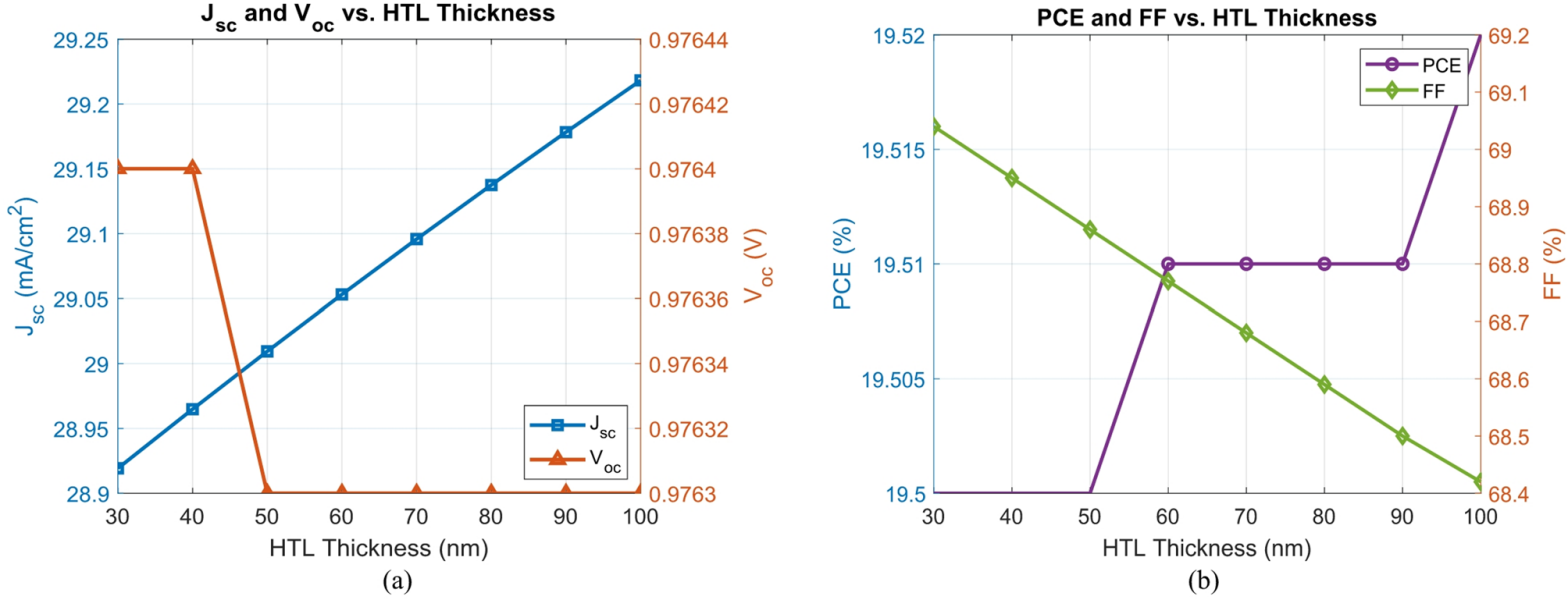
(a) ${V_{oc}}$ and ${J_{sc}}$versus PEDOT: PSS thickness, (b) *FF* and *PCE* versus PEDOT: PSS thickness.

These trends are shown in Fig. 7, which consists of two subplots. Figure 7(a) displays the evolution of ${J_{sc}}$ and ${V_{oc}}$ with increasing HTL thickness, while Fig. 7(b) illustrates changes in *PCE* and *FF*. The analysis suggests that while the performance is stable over the 30–100 nm range, thicknesses around 30–50 nm offer the most favorable trade-off between electrical conductivity and optical enhancement.

## Artificial intelligence-based performance prediction

Accurate prediction of OSC performance is crucial for guiding `material selection and device architecture optimization. Traditional methods, such as experimental fabrication or iterative numerical simulations are often time-consuming and computationally expensive. To overcome these limitations, AI offers a promising alternative, capable of learning complex, nonlinear relationships between device parameters and performance metrics from data^35^.

In this study, AI-based approaches are employed to predict two critical photovoltaic parameters, *PCE* and ${V_{oc}}$ based specifically on the structural configuration of the device. The main idea behind this method is to avoid running lots of simulations by figuring out the often tricky, nonlinear links between how the device is designed and how it performs. A dataset of 300 samples is generated using SCAPS-1D, covering various thickness values for three key layers in the OSC structure: the ETL, the active layer, and the HTL. Each data point shows the thickness of the three layers as inputs and the resulting *PCE* and ${V_{oc}}$ as outputs. Then, the data gets split by about 70% goes to training the model, while the remaining 30% is held back to test how well the model performs.

CNN and SVR are selected as the primary models due to their complementary strengths, while other machine learning approaches, such as Random Forest, XGBoost, and LSTM, also offer valuable insights as shown in Table 5. SVR provides high accuracy on small datasets and interpretable predictions, making it suitable for materials science applications where data is limited. CNN excels at automatically extracting complex features and generalizes well to unseen data, which is particularly effective for capturing nonlinear relationships between device structure and photovoltaic performance. Future work includes extending the benchmarking to Random Forest, XGBoost, LSTM, and other techniques to provide a broader comparison of AI approaches for organic solar cell performance prediction.

**Table 5:** Comparative illustrations for different AI techniques based on their key features, strengths, and limitations.

Model	Key Features	Strengths	Limitations
Support Vector Regression (SVR)	Maps nonlinear relationships using kernel functions^36^	High accuracy on small datasets; robust to overfitting; interpretable^37^	Struggles with very large datasets^38^
Artificial Neural Network (ANN)	modelling complex nonlinear relationships via hidden layers^39^	Learns complex nonlinear relationships; fault-tolerant; works with incomplete/noisy data; parallel processing^40^	Opaque decision-making; requires large data and computation; network design trial-and-error^40^
Convolutional Neural Network (CNN)	Automatically extracts relevant features through convolutional and pooling layers^41^	Excellent feature extraction; captures complex nonlinear relationships; strong generalization^42^	High computational cost; lower interpretability and explainability^41^
Random Forest (RF)	Collection of many decision trees^43^	Handles complex nonlinear relationships and feature interactions; manages unbalanced and missing data; mitigates overfitting through ensemble learning; provides high generalization and predictive accuracy^44,45^	Requires substantial amounts of high-quality training data^45^
Extreme Gradient Boosting (XGBoost)	Scalable tree-boosting algorithm designed for high performance, adaptability, and mobility^46^	High flexibility, scalability, and computational efficiency; reduces overfitting^47^	Can overfit if hyperparameters are not tuned; hard to interpret with many trees; longer training time^48^
Long Short-Term Memory (LSTM)	Captures temporal dependencies in sequential data^49^	Effective for time-series and sequence modelling; can model complex temporal patterns^49^	Requires large datasets; prone to overfitting; performance sensitive to proper training and regularization^49^

This AI framework serves as a predictive tool for OSC performance, allowing researchers to quickly evaluate how different layer setups affect results without the need for time-consuming simulations. The next sections describe the development, structure, and evaluation of two supervised learning models: a Convolutional Neural Network (CNN) and Support Vector Regression (SVR). These models are chosen to compare deep learning techniques with more traditional machine learning methods for predicting device performance. Although the dataset size is relatively modest, which increases the risk of overfitting in deep learning models such as CNNs, this limitation is addressed by incorporating model-to-model comparisons (CNN vs. SVR) and by reporting quantitative performance metrics, including Root Mean Square Error (RMSE). These steps strengthen the robustness and transparency of the analysis. The CNN can capture complex nonlinear relationships within the data, while the SVR serves as a complementary benchmark, highlighting how conventional machine learning approaches can achieve comparable predictive accuracy when data availability is limited.

### Convolutional neural network (CNN) model

**Fig. 8:**
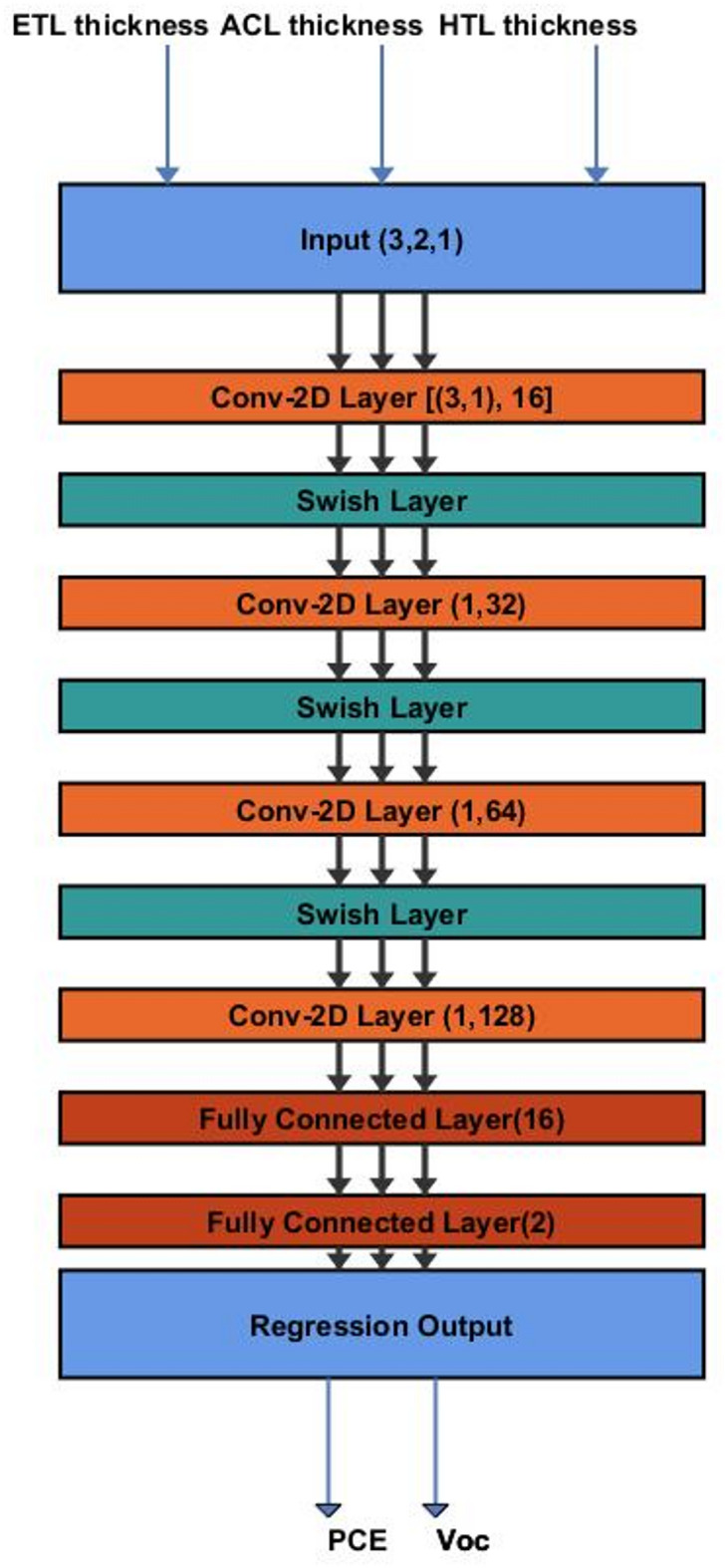
Architecture of the Convolutional Neural Network (CNN) used for OSC performance prediction.

A CNN model is developed and trained on data from SCAPS-1D simulations to predict OSC performance based on structural design. Although CNNs are commonly used for image tasks, they also perform well in regression problems when adapted correctly^50^. In this work, a tailored CNN architecture is designed to predict two continuous photovoltaic parameters *PCE* and ${V_{oc}}$ using three key input features: the thicknesses of ETL, the active layer, and HTL. The CNN model has 11 layers. It starts with an input layer that takes the three numerical features. Then comes a 2D convolutional layer, which uses filters to pick up patterns and relationships between these inputs. The Swish activation function runs through the network because it’s smooth and non-monotonic, which helps gradients flow better and improves how well the model predicts^51^. The network finishes with fully connected layers and a regression output that predicts both *PCE* and ${V_{oc}}$ at the same time. Figure 8 shows the architecture.

**Fig. 9:**
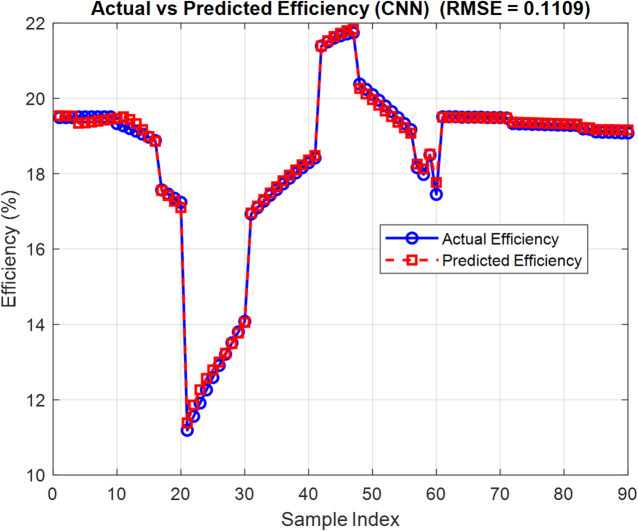
Predicted vs. actual power conversion efficiency (*PCE*) using CNN.

**Fig. 10:**
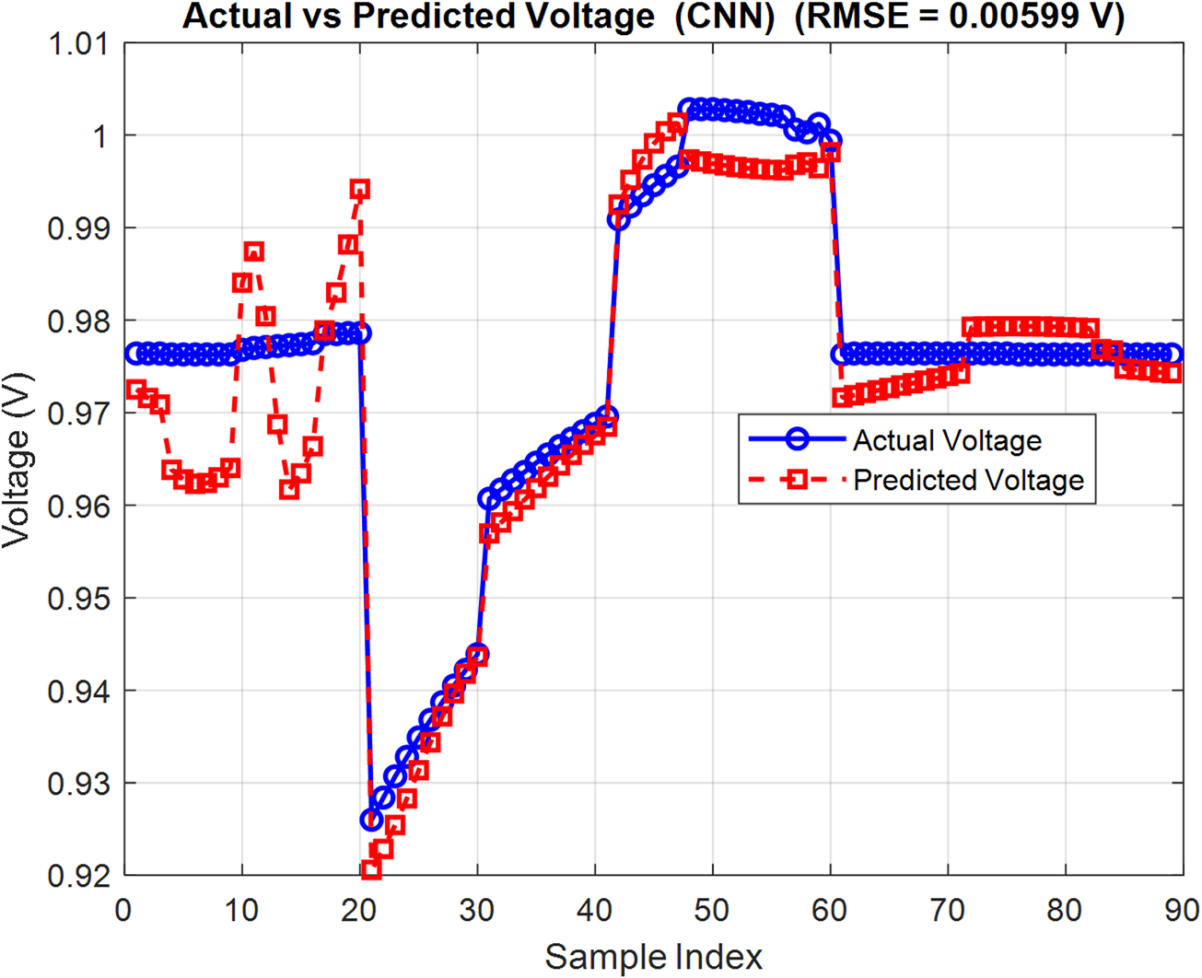
Predicted vs. actual open-circuit voltage (${V_{oc}}$) using CNN.

**Fig. 11:**
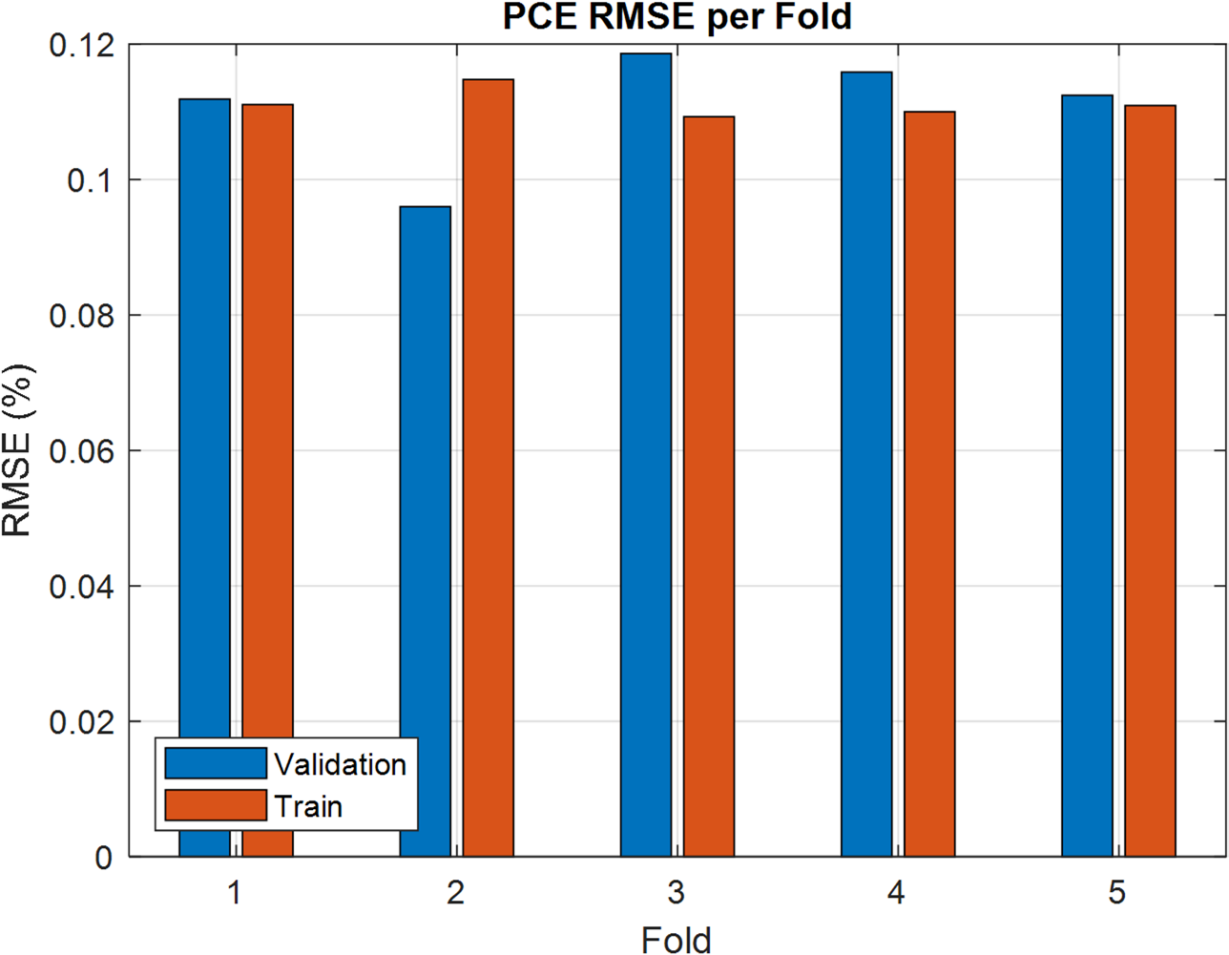
RMSE for PCE per Fold.

**Fig. 12:**
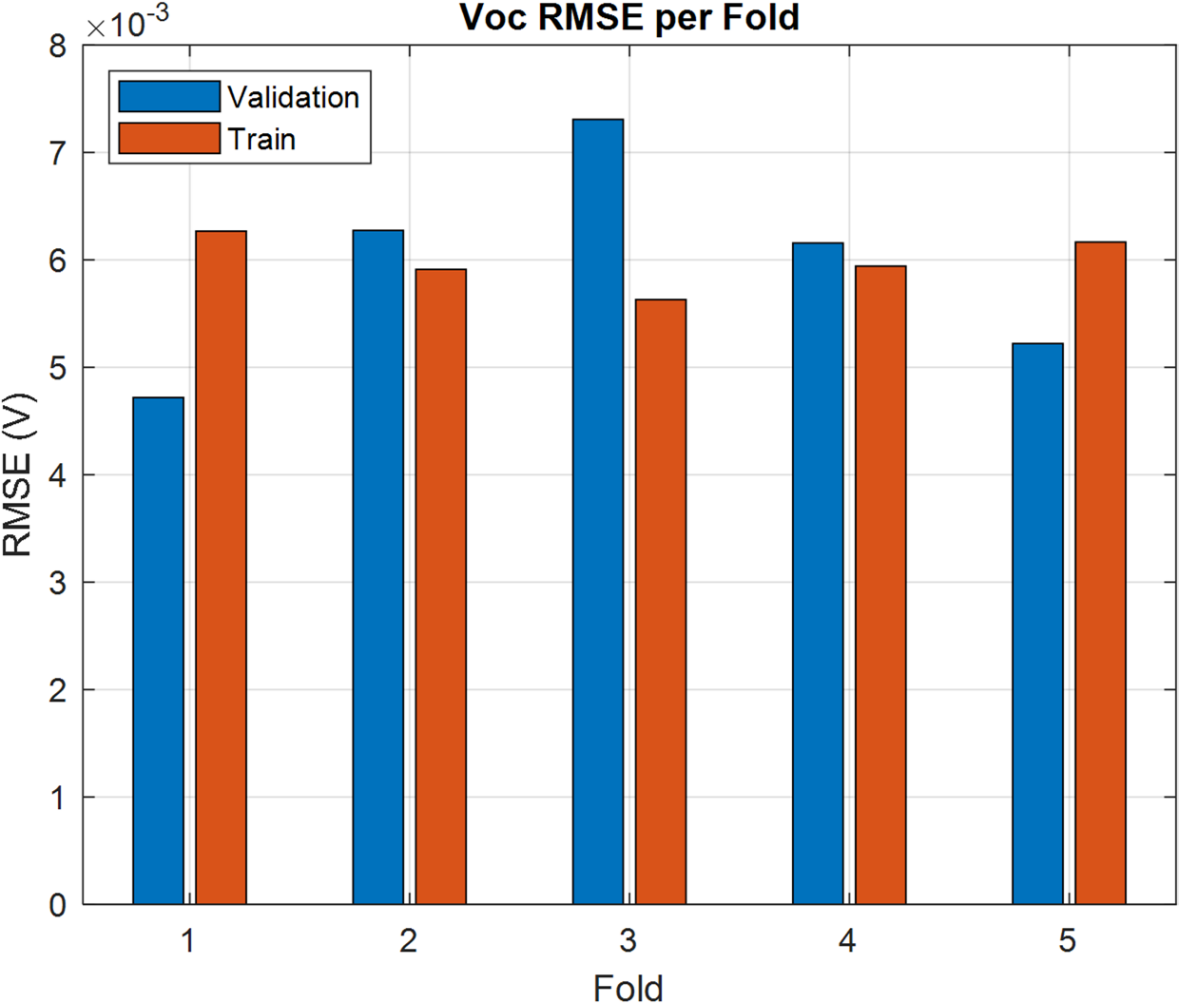
RMSE for V_oc_ per Fold.

Training runs for 500 epochs. Hyperparameters like learning rate, batch size, and kernel size are adjusted using grid search to identify the best settings. On the test data, predictions are quite accurate, with RMSE values around 0.11 for *PCE* and 0.006 for ${V_{oc}}$. These results underscore the model’s excellent generalization ability and reliability. Figures 9 and 10 show how predicted and real values match up closely, proving the CNN’s strength in predicting OSC performance quickly and reliably. In order to ensure that CNN can effectively address the issue of overfitting, the k-fold is examined as shown in Figs. 11 and 12. The bar charts display training and validation RMSE for PCE and ${V_{oc}}$ respectively. For PCE, the mean RMSE and Mean Absolute Error (MAE) are 0.1109 and 0.081576, with a mean coefficient of determination ( R²) of 0.99553. For ${V_{oc}}$, the mean RMSE and MAE are 0.00599 V and 0.00472 V, with a mean R^2^ of 0.8882. The figures highlight consistent performance across all folds, demonstrating that the model predictions remain robust and reliable throughout the dataset.

### Support vector regression (SVR)

In parallel with the CNN approach, Support Vector Regression (SVR) is employed as an alternative machine learning method to predict *PCE* of OSCs based on their structural parameters. SVR, an extension of the Support Vector Machine (SVM) algorithm for regression tasks, is particularly well-suited for modelling nonlinear relationships in small to medium-sized datasets, an important consideration in materials science applications where extensive data is often limited^52,53^.

The fundamental objective of SVR is to identify a function $f{\mathrm{~}}\left( {x,\alpha } \right)$ that best approximates the relationship between the input features and the target output, while tolerating a predefined margin of error. The model relies on kernel functions to project the input data into a higher-dimensional space where complex patterns can be linearly separated. In this study, a Radial Basis Function (RBF) kernel is selected due to its flexibility and effectiveness in capturing nonlinear dependencies^54^. The SVR formulation used is expressed as:

4 $$f{\mathrm{~}}\left( {x,\alpha } \right)={\mathrm{~}}\mathop \sum \limits_{{i=1}}^{{{N_d}}} {\alpha _{i{\mathrm{~}}}}K{\mathrm{~}}\left( {{x_i},{x_j}} \right)+b$$

Where $K{\mathrm{~}}\left( {{x_i},{x_j}} \right)$ is the kernel function, ${\alpha _{i{\mathrm{~}}}}$ are the model weights, and b is the bias term adjusting the regression output. This formulation enables the model to learn complex, nonlinear mappings between the device architecture specifically, the thicknesses ETL, the active layer, and HTL and the resulting *PCE* and ${V_{oc}}$.

**Fig. 13:**
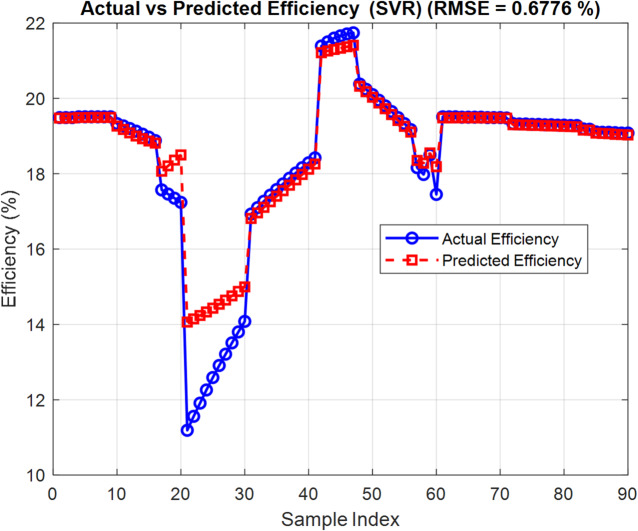
Conceptual illustration of the SVR model used for *PCE* prediction.

**Fig. 14:**
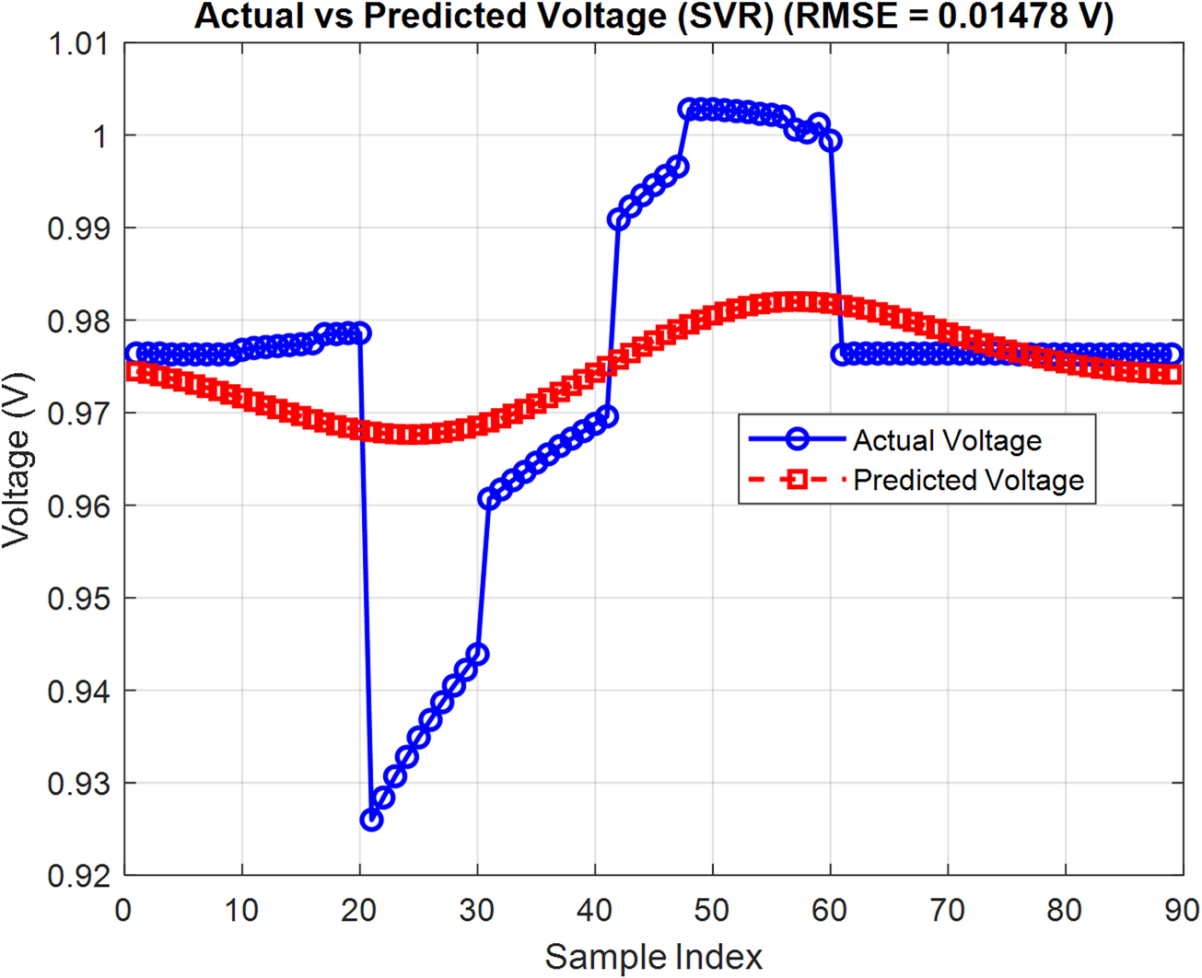
Predicted vs. actual *PCE* values using the SVR model.

The SVR model is trained using the same dataset as the CNN model, ensuring a consistent basis for comparison. Following training and hyperparameter optimization, the SVR model achieved RMSE of 0.6776 on the test set. Although this is higher than the CNN’s performance (RMSE = 0.1109), the SVR results still reflect its capacity to generalize the structural-performance relationship of OSCs with reasonable accuracy. Figures 13 and 14 illustrate the SVR model structure and the comparison between predicted and actual *PCE* values, respectively. The observed trend alignment suggests that SVR remains a valuable predictive tool, particularly in cases where computational simplicity and interpretability are prioritized over precision.

### Model comparison

To evaluate the predictive capabilities of various AI models in estimating the performance of OSCs, a comparative study is carried out involving CNN and SVR. All models are trained on the same dataset comprising 300 samples generated using SCAPS-1D simulations. Each sample includes three input features representing ETL, active layer, and HTL thickness. The target outputs for model training and evaluation are *PCE* and ${V_{oc}}$.

**Fig. 15:**
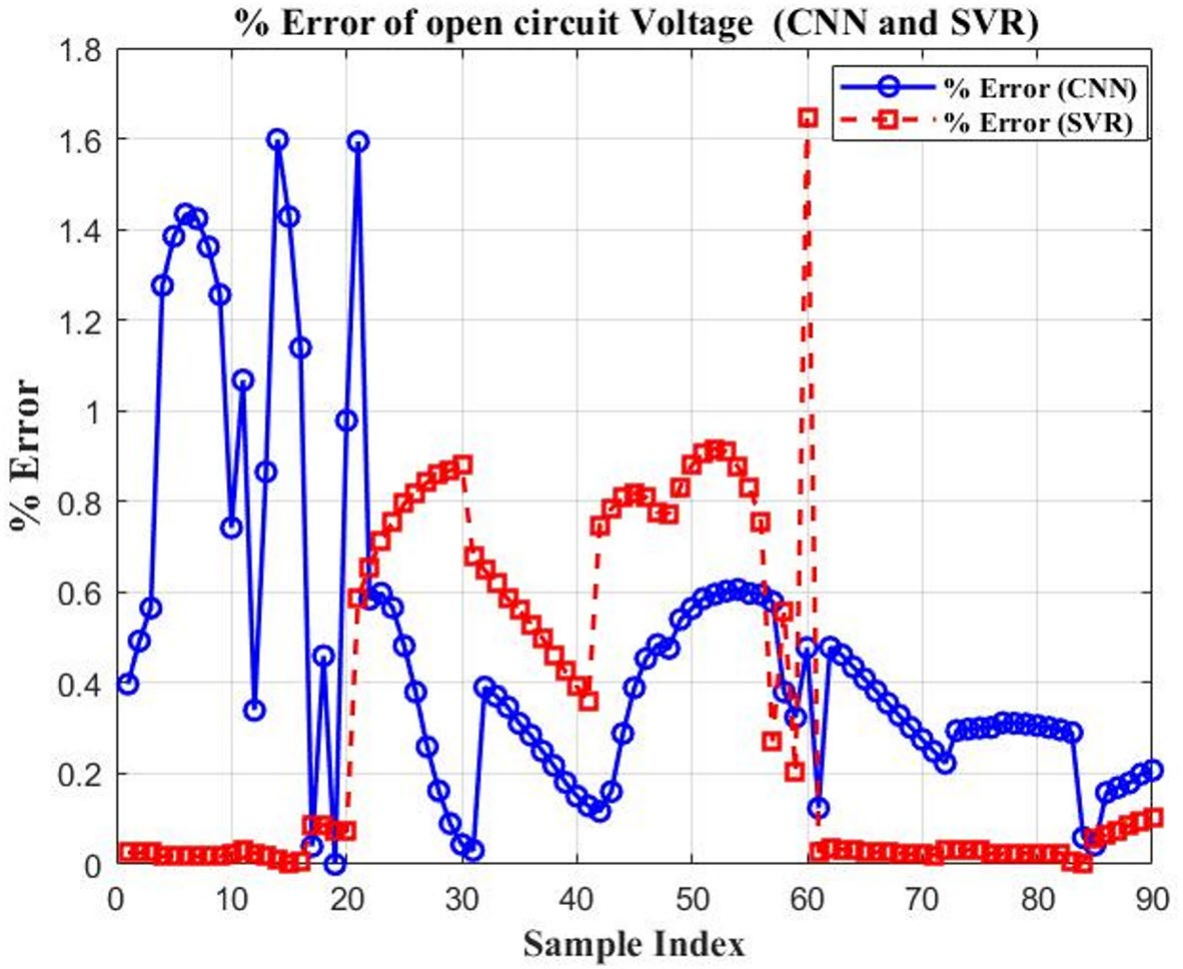
The percentage output error of two proposed AI for different tested samples ($${V_{oc}})$$.

**Fig. 16:**
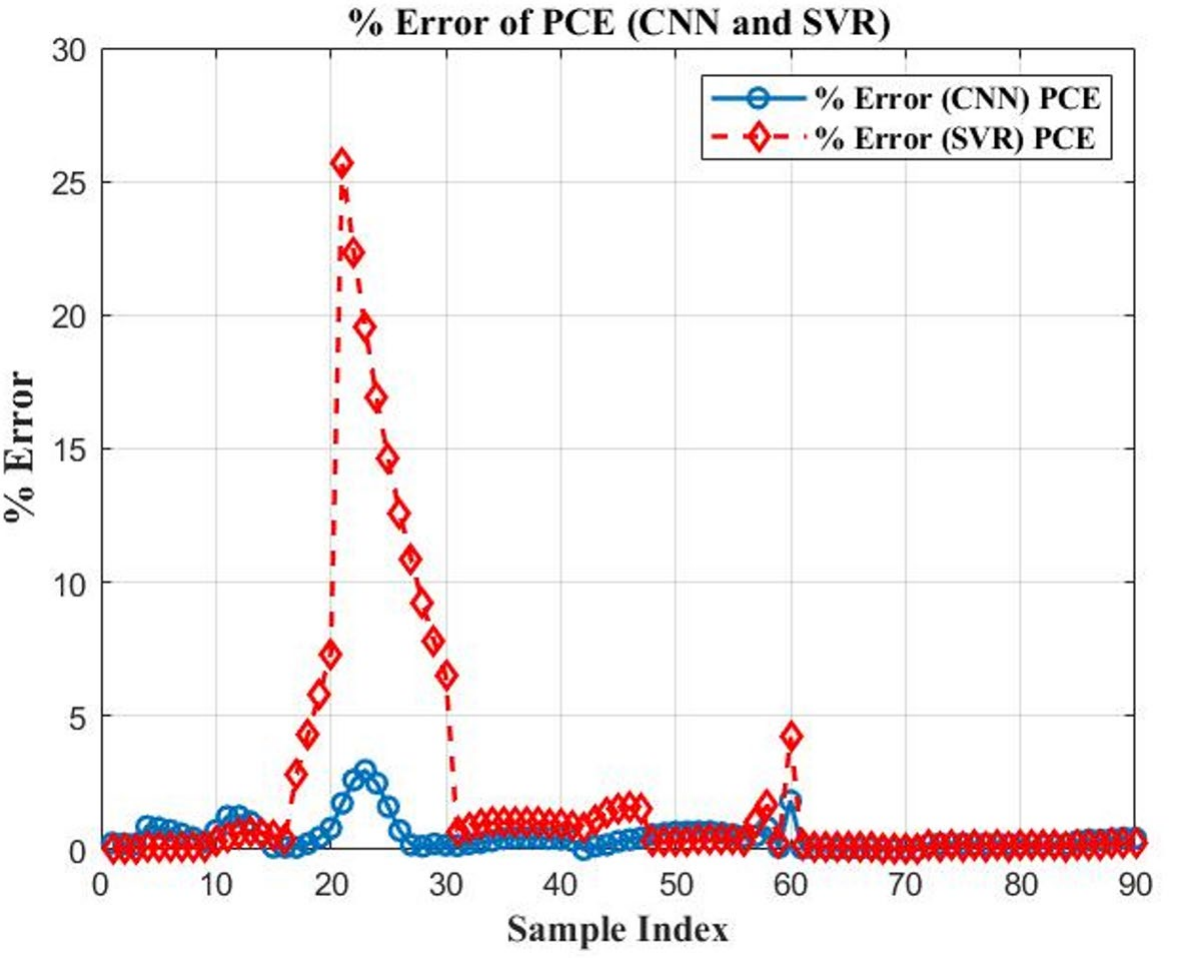
The percentage output error of two proposed AI for different tested samples ($$PCE)$$.

**Fig. 17:**
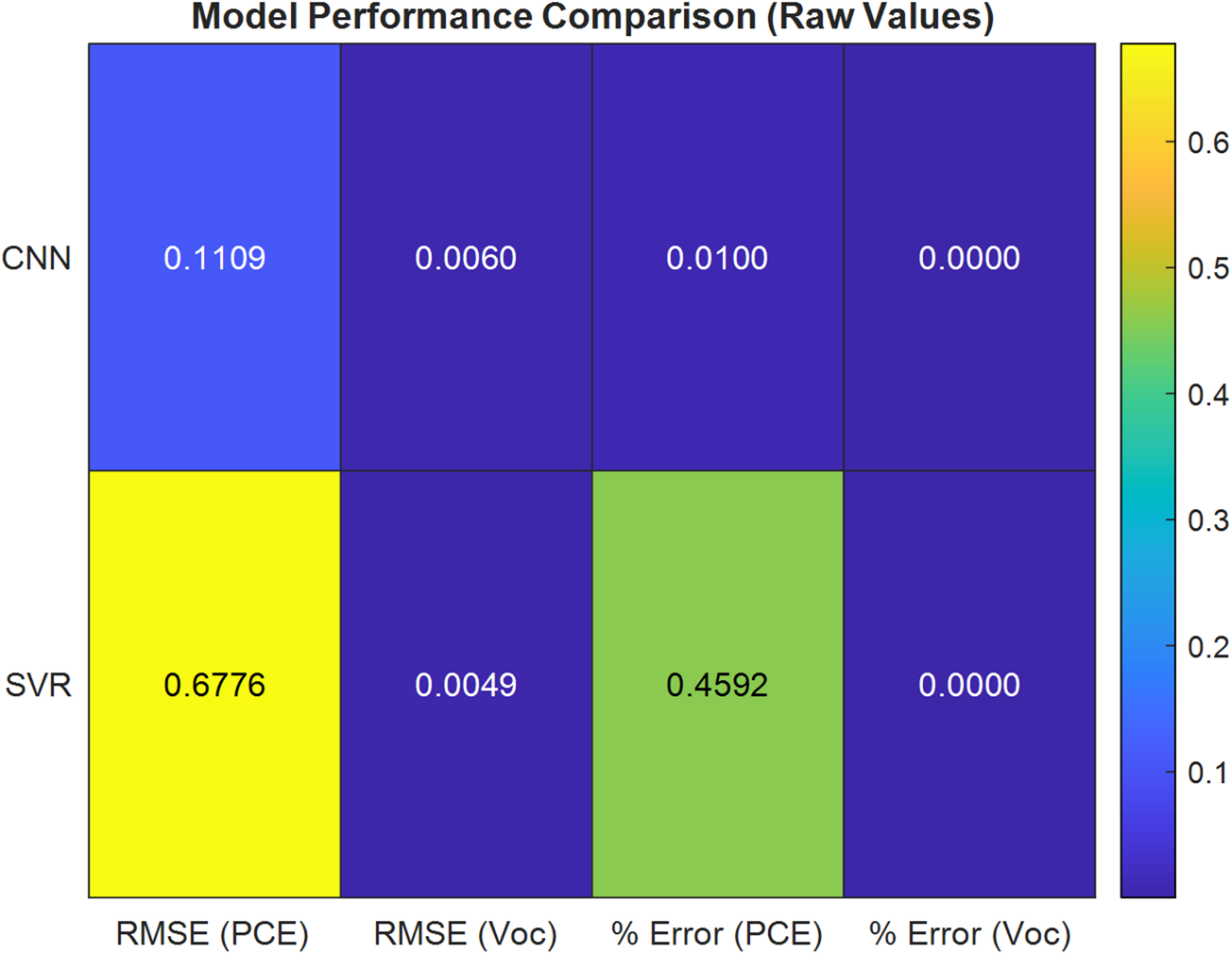
Model performance evaluation based on RMSE and prediction error (%).

Figures 15 and 16 illustrate the percentage error of *PCE* and the ${V_{oc}}$ of CNN and SVR. The two proposed AI are compared in Table 6 based on the difference between their RMSE and maximal MAE. The performance of the models is assessed using two key metrics: RMSE and percentage prediction error. The results are visually illustrated in Fig. 17, which provides a direct comparison of model accuracy.

**Table 6:** Comparison between three proposed artificial intelligent.

The distinctions	(${V_{oc}}$) CNN	(${V_{oc}}$ ) SVR	(PCE) CNN	(PCE) SVR
RMSE	0.0060	0.0049	0.1109	0.6776
Maximum MAE	0.000035488	0.00002	0.01230398	0.45920498

## Sustainability impact and alignment with SDGs

**Fig. 18:**
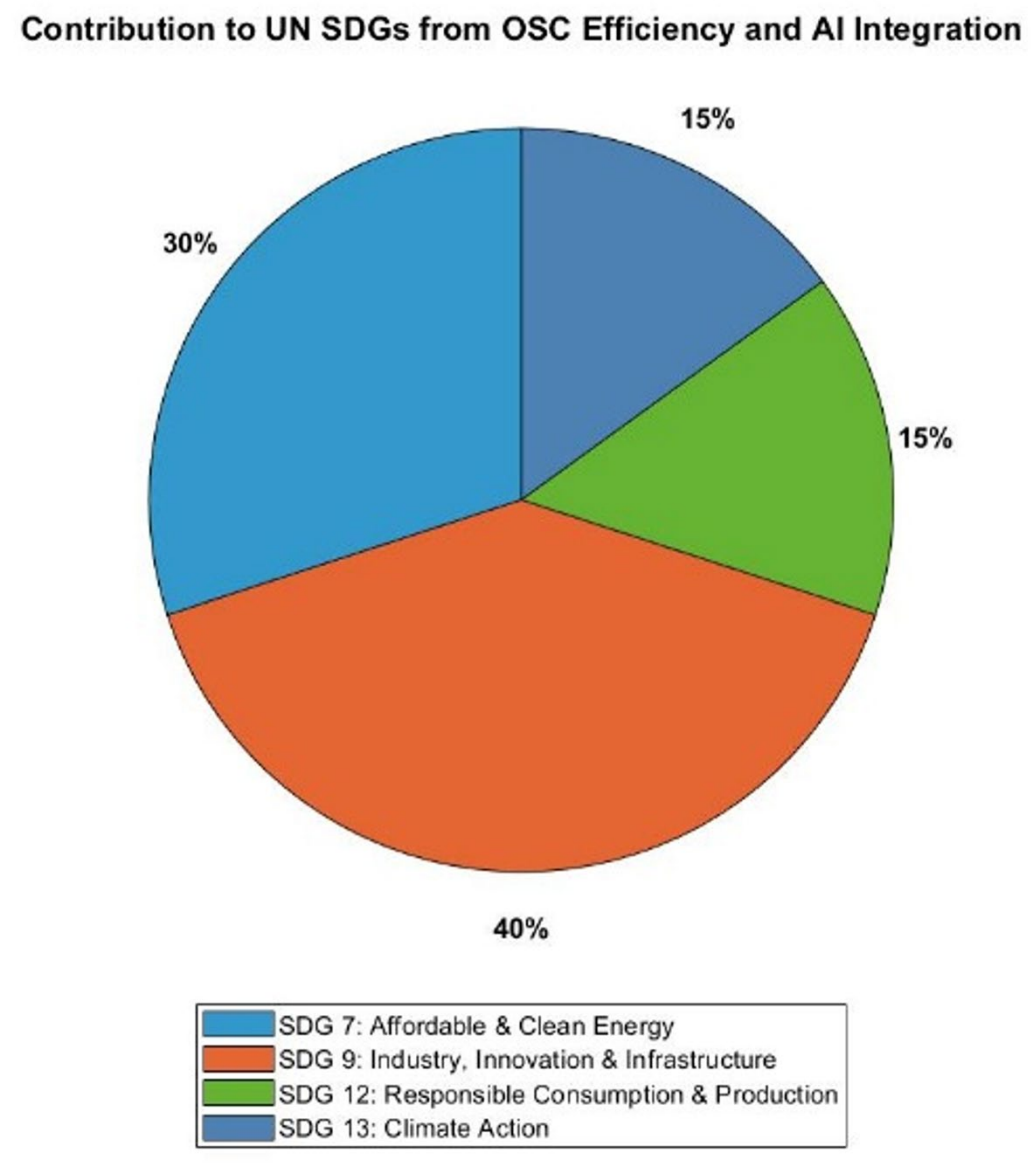
The contribution of the research work to SDGs.

The pie chart, shown in Fig. 18, indicates the extent to which this research contributes to four critical UN Sustainable Development Goals (SDGs). The percentages are based on measurable enhancements from both AI refinement and simulation. The pivotal role of AI—particularly CNNs in optimizing device performance is indicated by the fact that a dominant 40% is associated with SDG 9: Industry, Innovation, and Infrastructure. The CNN model reduced prediction errors for *PCE* by over 97% and cut RMSE by more than 83% compared to SVR, enabling more intelligent and efficient design processes. SDG 7: Affordable and Clean Energy accounts for 30%, driven by a 61.9% boost in solar cell efficiency—from 12.04% to 19.50%, which allows for greater clean energy output from the same area. SDG 12: Responsible Consumption and Production accounts for 15% of the target, as increased efficiency reduces the material footprint by necessitating a smaller active area to produce the same amount of energy. SDG 13: Climate Action is responsible for an additional 15% of the reduction in environmental impact, which is attributable to the reduced material and energy consumption and enhanced performance of the device throughout its lifecycle. These allocations illustrate the potential of an AI-generated and AI-refined optimization strategy to significantly contribute to the achievement of global sustainability objectives by means of advanced solar cell engineering. The percentages reported are derived from a weighted approach quantitative structure based on the authors’ perspective on the contribution and impact of each SDG in the study. Each improvement observed in this study is assigned by a weight proportional to its relative magnitude.

## Conclusions

This study demonstrates the power of integrating numerical simulation with machine learning to optimize the performance of OSCs. SCAPS-1D is used to study how different materials and layer thicknesses affect efficiency in a model OSC structure: ITO/PEDOT: PSS/PBDB-T: IT-M/PFN-Br/Al. The simulation matches experimental results well, showing it predicts device behavior accurately. Among ETL materials tested, PFN-Br gives the highest simulated efficiency at 12.04%. This likely comes from its good energy alignment and low-resistance contacts. Optimizing the thickness finds that a 5 nm PFN-Br layer hits the sweet spot between good charge extraction and keeping recombination losses low. For the active layer, a thickness of 300 nm gives the best results, capturing more light without making charge transport less efficient. This setup reaches a peak simulated *PCE* of 19.50%. Among HTL tested, PEDOT: PSS stands out as the most reliable option.

To speed up the optimization process, machine learning models specifically CNN and SVR, are used to predict key performance metrics like *PCE* and ${V_{oc}}$ from structural input parameters. Among the two, CNN delivers higher predictive accuracy, highlighting its strength in capturing the complex nonlinear patterns found in OSC design.

For *PCE*, CNN achieves an RMSE of 0.1109, while SVR records 0.6776, representing an 83.6% reduction in RMSE. Similarly, the percentage error for the *PCE* prediction drops from 0.4592% (SVR) to 0.01% (CNN), a 97.8% improvement. For ${V_{oc}}$, the SVR reduces RMSE from 0.0060 (CNN) to 0.0049. In terms of ${V_{oc}}$ percentage error, SVR performs better with 0.000035488% compared to CNN’s 0.00002426%. CNN appears slightly worse (an increase of ~ 22.4%). Overall, CNN shows a strong advantage, particularly in modelling *PCE*, underscoring its ability to capture the nonlinear behavior inherent in OSC structures.

Combining physics-based simulations with data-driven modelling, this approach offers a practical and scalable way to speed up the optimization of next-generation OSCs. It improves both the pace of device design and the accuracy of performance predictions. By merging these methods, the process from design to prototype becomes faster, while also paving the way for smarter, AI-assisted tools in photovoltaic research. As a result, this work lays a strong foundation for sustainable solar technologies that can help deliver affordable clean power (SDG 7), foster innovative industry (SDG 9), minimize resource use (SDG 12), and support climate action (SDG 13).

## Data Availability

All data generated or analyzed during this study are included in this article.
